# The Impact of Health Consciousness on the Association Between Walking Durations and Mental Health Conditions After a Disaster: a Cross-Sectional Study

**DOI:** 10.1186/s40798-020-00259-6

**Published:** 2020-07-16

**Authors:** Yusuke Utsumi, Harumi Nemoto, Naoki Nakaya, Tomohiro Nakamura, Naho Tsuchiya, Akira Narita, Mana Kogure, Tomomi Suzuki, Moe Seto, Mitsuaki Katayanagi, Junko Okuyama, Atsushi Sakuma, Nami Honda, Yumi Sugawara, Halley Kaye-Kauderer, Yuta Takahashi, Akemi Kayama, Yoshihisa Kakuto, Masahiro Kohzuki, Atsushi Hozawa, Ichiro Tsuji, Hiroaki Tomita

**Affiliations:** 1 Department of Disaster Psychiatry, Graduate School of Medicine, Tohoku University, 2-1 Seiryo-Machi, Aoba-ku, Sendai, 980-8573 Japan; 2 Miyagi Psychiatric Center, Mubanchi, Tekurada, Natori, 981-1231 Japan; 3 Department of Disaster Psychiatry, International Research Institute of Disaster Science, Tohoku University, 2-1 Seiryo-Machi, Aoba-ku, Sendai, 980-8573 Japan; 4 Department of Preventive Medicine and Epidemiology, Tohoku Medical Megabank Organization, Tohoku University, 2-1 Seiryo-Machi, Aoba-ku, Sendai, 980-8573 Japan; 5 Department of Psychiatry, Tohoku University Hospital, 1-1 Seiryo-Machi, Aoba-ku, Sendai, 980-8574 Japan; 6 Department of Psychiatry, Graduate School of Medicine, Tohoku University, 2-1 Seiryo-Machi, Aoba-ku, Sendai, 980-8575 Japan; 7 Department of Public Health, Graduate School of Medicine, Tohoku University, 1-1 Seiryo-machi, Aoba-ku, Sendai, 980-8574 Japan; 8 Department of Medical Education, Icahn School of Medicine at Mount Sinai, 1 Gustave L. Levy Pl, New York, NY 10029 USA; 9 Department of Occupational Therapy, Tohoku Bunka Gakuen University, 6-45-1 Kunimi, Sendai, 981-8551 Japan; 10 Department of Internal Medicine and Rehabilitation Science, Graduate School of Medicine, Tohoku University, 2-1 Seiryo-Machi, Aoba-ku, Sendai, 980-8575 Japan

**Keywords:** Physical activity, Walking habits, Health consciousness, Motivation, Optimism, Mental health, Depressive symptoms, Posttraumatic stress reaction, Disaster, the Great East Japan Earthquake

## Abstract

**Background:**

In communities affected by a disaster, walking can be a feasible form of physical exercise to improve physical and mental health conditions. However, there is limited evidence to support relationships between walking habits and mental health conditions in post-disaster settings. Cross-sectional epidemiological data obtained from a questionnaire survey (conducted in October 2017) of a community affected by the 2011 Great East Japan Earthquake (GEJE) was analyzed to evaluate the relationships.

**Methods:**

Participants included individuals over 20 years of age (*N* = 718) from Shichigahama town in Miyagi prefecture, whose houses were significantly damaged by the GEJE. Their mental health conditions were assessed by the Kessler Psychological Distress Scale (K6), the Center for Epidemiologic Studies Depression Scale (CES-D), and the Impact of Event Scale-Revised (IES-R). Additionally, the questionnaire asked the participants spent duration walking on average and their walking purpose by the following items: (1) longer than 60 min per day, (2) between 30 and 60 min per day, or (3) less than 30 min per day, and whether they walked to maintain healthy living habits (health-conscious walkers) or merely for transportation without considering health consequences (non-health-conscious walkers). These information and mental health indicators were analyzed using analysis of covariance (ANCOVA).

**Results:**

Among the three walking duration groups of health-conscious walkers, there were significant differences in CES-D and K6 scores (*p* = 0.01 and *p* = 0.04), but not in IES-R scores, considering age, gender, and alcohol drinking habits as covariates. CES-D score was significantly higher among short walkers (*p* = 0.004). Among the three walking duration groups of non-health-conscious walkers, there were significant differences in avoidance symptoms, the subdomain of IES-R (*p* = 0.01), but not in CES-D, K6, and total IES-R scores, considering the variants.

**Conclusion:**

Our study suggests that walking durations may positively affect mood, but not PTSR, only when walking is performed with the purpose of maintaining healthy living habits. Walking durations were negatively associated with avoidance symptoms among non-health-conscious walkers in the community affected by the GEJE, indicating that the disaster may have had a long-lasting impact on walking habits.

## Key Points

- Among the population who walked for health, walking durations were negatively associated with depressive symptoms but not with posttraumatic stress reactions.
- Among the population who walked for other reasons besides health, walking durations were negatively associated with the avoidance symptoms of posttraumatic stress reaction.
- Encouraging walking for health reasons and re-arranging the surrounding environment of the communities affected by a disaster may help to improve their mental health conditions.

## Background

On March 11th, 2011, the northeastern coast of Japan was devastated by the magnitude 9.0 Great East Japan Earthquake (GEJE) and the tsunami that followed. Previous studies indicated that a wide range of stressors, such as house damages caused by the disaster, were associated with mental health consequences, including depressive states and posttraumatic stress reactions (PTSR) [1–6].

In ordinary settings, general physical activities [7–16], including walking [17–22], are positively associated with mental health and well-being and negatively associated with depressive symptoms regardless of age and gender. However, the relationship between physical activities and mental health conditions has not been well characterized in post-disaster settings with an exception which surveyed the area affected by the GEJE and found that decreased physical activity was significantly associated with increased levels of depressive states among elderly people aged ≧ 65 [23, 24]. Likewise, evidence suggests that physical activities such as yoga, aerobics, and resistance training have positively effects on people suffering from PTSR. However, due to the limited number of RCTs, it is unclear the optimal frequency, intensity, type, and time (FITT) of physical activity needed to improve the well-being of people with PTSR [25]. Additionally, there is limited research investigating the relationship between physical activity and PTSR in communities affected by disasters.

It is crucial to evaluate the associations between physical activities and PTSR because persistent avoidance of stimuli is one of the major symptoms of PTSR, along with intrusion symptoms, negative alterations in cognition and mood, and alterations in hypervigilance and reactivity [26]. The avoidance may theoretically deter people suffering from PTSR from walking outside in surrounding areas where they anticipate stimuli by seeing signs or landscapes that evoke trauma-related memories.

It is especially important to evaluate associations between walking habits and mental health conditions in a post-disaster setting because walking remains a feasible form of exercise for affected individuals when a disaster damages other exercise facilities within a community. On the other hand, it is noteworthy that people walk not only for the direct purpose of promoting their health but also for the purpose of dealing with daily tasks like business endeavors, shopping, or chores. While the purpose of walking may theoretically impact the effect of walking on health conditions, this purpose has rarely been investigated in previous studies. One study that explored this question looked at the relationship between the purpose of walking and health conditions in adults aged 60–64 years. This study revealed that participation in leisure-time physical activities and walking for pleasure were positively correlated with mental health and well-being, whereas total levels of free-living physical activities were not [27]. In addition, people with PTSD and depressive symptoms may face considerable barriers to engaging in regular physical activity, similar to those with other mental health disorders (for example, a lack of motivation or optimism) [28–30]. Motivation and optimism (positive expectations of the future) may increase an individual’s desire to participate in exercise programs and modify their health behaviors, ultimately leading to improved mental health and well-being.

To figure out the above unelucidated questions regarding walking habits and mental health conditions in communities affected by a disaster, the current study aims to investigate the difference in mental health conditions, such as depressive state and PTSR, among people who had long or short durations of daily walking in post-disaster settings, and how they differ among populations who walked primarily with or without the purpose of maintaining a good health condition. The study also intended to examine the difference in emotional pain when seeing signs relevant to a disaster among the subgroups with different walking habits.

## Methods

### Study Design, Settings, and Participants

The present study is based on the Shichigahama Health Promotion Project conducted in partnership between Tohoku University and Shichigahama town, located on the coast of Miyagi Prefecture and previously devastated by the GEJE [2–6]. This project was initiated to improve the mental health and well-being of communities living in Shichigahama town. It began with outreach activities for individuals living in evacuation camps immediately after the disaster and later expanded to personal mental health care and mental health promotions, including mental health workshops for people who moved to prefabricated temporary housings. In addition, annual health surveys of the affected population of the town were conducted since 2011, along with follow-up support programs. Among the total residents at the onset of the GEJE (approximately 22,000; current residents, approximately 19,000), there were approximately 2800 identified as people whose houses were totally collapsed or majorly damaged based on public evaluations of the damage. The initial health survey was conducted in October 2011, and subsequent annual surveys have been conducted since then.

In this study, cross-sectional analysis was conducted using data obtained through self-administered questionnaires of the 7th survey conducted in 2017 as well as additional questions focused on walking habits and living environments. The questionnaires were sent and collected via the postal service. The inclusion criteria of the survey were all residents who lived in Shichigahama town at the time of onset of the GEJE and whose houses were severely damaged by the GEJE according to the standard for the public disaster-victim certificate. The inclusion criteria of the current study were individuals over 20 years of age who completed questionnaires regarding mental health, well-being, and walking habits. As for the informed consent process, we declared in the cover letter of the survey that we regarded responses to the questionnaire as consent to participate in the study. Participants were informed that they were free *not* to participate in the study, with absolutely no negative consequences in any way. Also, participants were told that the findings of this study would be published without any identifying personal information. All personal information, including individual names and addresses, were omitted from the data and substituted with unique codes as identification. The anonymized data was subjected to the subsequent analysis. The study protocol was reviewed and approved by the Ethics Committee of Tohoku University Graduate School of Medicine.

The questionnaires were sent to 3 to 6 age (14 subjects, 9 respondents, response rate = 64%), elementary school students to junior high school students (124 subjects, 84 respondents, response rate = 68%), high school student equivalent (53 subjects, 36 respondents, response rate = 68%), 18 to 19 age (46 subjects, 27 respondents, response rate = 59 %), adults (2489 subjects, 1334 respondents, response rate = 54%), and total (2726 subjects, all of whom fitted the inclusion criteria, and 1490 responded, response rate = 55%). Among them, 718 completed all of the questionnaires for mental health conditions, walking habits, and living environment, which were subjected to the subsequent analysis.

### Questionnaires

The questionnaires of Shichigahama Health Promotion Project survey consisted of the following items: clinical and sociodemographic information (age, sex, marital status, education level, and socioeconomic status), lifestyle behaviors (smoking and alcohol drinking habits), social interactions, social capital, and general health conditions (present medical history, body weight, and height). Regarding alcohol drinking habit and smoking drinking habit were asked as follows. The participants were asked, “Do you drink alcohol?” or “Do you smoke?” and select from “yes” or “no” for the question. The questionnaire also requested detailed information related to how participants experienced the earthquake and tsunami, including the subjects’ location at the time of onset of the disaster and evacuation routes, damage to their homes, and loss of family members and close acquaintances.

In order to examine mental health and well-being, psychological distress scores ranging from 0 to 24 were assessed using the Japanese version of the Kessler 6-items scale (K6) [31–34]. Psychological distress scores of ≥ 9 were classified as a distressed condition. The Center for Epidemiologic Studies Depression Scale (CES-D) was administered to all of the participants to evaluate their depressive symptoms. Each of the 20 items on the CES-D was rated on a 4-point scale ranging from 0 to 3, which referred to how often the respondent experienced depressive symptoms during the past week. The sum of the scores ranged from 0 to 60, and scores equal to or above the cut-off score of 16 indicated a depressive state [35]. Impact of Event Scale-Revised (IES-R), consisting of 22 items with a 5-point scale (from 0 to 4), was used for evaluating the severity of PTSR during the previous 7 days. The scores equal to or above the cut-off score of 25 suggested the presence of PTSR [36]. The three subcategories of PTSR symptoms were also examined using eight items for re-experiencing items, eight items for avoidance, and six items for hyperarousal.

The questionnaire specifically asked how many minutes the subjects spent walking on average by selecting one of the following items: (1) longer than 60 min per day, (2) between 30 and 60 min per day, or (3) less than 30 min per day, and classified into “long walkers,” “moderate walkers,” or “short walkers,” respectively, in the subsequent analyses. Additional questions were added after the following indication; “We would like to ask your walking habit and living environment. Please select the most appropriate answer for the following statement”. Participants were asked to select from “yes” or “no” for the question; “The reason for your daily walking is for your health?”. The participants who selected “yes” or “no” were classified as “health-conscious walkers” or “non-health-conscious walkers” in the subsequent analysis. Emotional pain related to observed landscape destruction is likely to be a factor that will impact walking behavior in post-disaster settings. Therefore, participants were asked to select from “Not applicable,” “not completely applicable,” “Somewhat applicable,” and “Applicable,” for the statement to describe their condition; “I feel emotional pain to see signs relevant to a disaster or landscape of the sea after the disaster.”

### Statistical Analyses

Differences in the distribution of age (equal to/higher than or younger than 65), gender (male or female), number of family members (1 to 9), residential status (living in the same place as before the GEJE, living in a different place, not due to the disaster, post-disaster publicly funded rental accommodation, houses of relatives or friends, prefabricated post-disaster public housing, newly built houses in the relocated high-land area, newly built houses outside of the relocated high-land area, or others), economic status (extremely difficult, difficult, a little difficult, or fine), alcohol and tobacco consumptions (alcohol drinking habit or non-alcohol drinking habit and smoking habit or non-smoking habit), purpose of walking (health consciousness walkers or non-health-conscious walkers), CES-D score-based depressed state (equal to/greater than or less than the cut-off point of 16), K6 score-based psychological pain (equal to/greater than or less than the cut-off point of 9), IES-R score-based PTSR (equal to/greater than or less than the cut-off point of 25), and among the three walking duration groups, long walkers, moderate walkers, and short walkers, were examined using a Chi-square test. Mental health indicators (CES-D scores, K6 scores, and IES-R scores) of all participants, health-conscious walkers, and non-health-conscious walkers were also compared as continuous variables among the three walking duration groups using analysis of covariance (ANCOVA). In addition, as subtests, the *p* values for walking durations adjusted by age, gender, and alcohol drinking habits were analyzed by multiple logistic regression model, because these factors showed significant differences in the distributions among the three groups in the above Chi-square analysis. Likewise, the differences in emotional pain to see signs relevant to a disaster or landscape of the sea in the living environment after the disaster were examined among the subgroups regarding the walking habits using ANCOVA, using gender, age, and alcohol drinking habits as covariates. All statistical analyses were conducted using EZR version 1.37, and a *p* value of < 0.05 was considered statistically significant [37].

## Results

### Analyses of All Participants

In the analyses of all participants, distributions of age, gender, and alcohol drinking habits were significantly different among the three walking groups—long walkers (> 60 min), moderate walkers (30–60 min), and short walkers (< 30 min) (*p* = 0.03, *p* < 0.001, and *p* = 0.03, respectively), and the proportion of elderlies (age ≧ 65), females, and alcohol drinkers were more substantial among the subgroups who walked shorter. The distribution of the subpopulation who walked primarily with or without the purpose of maintaining good health conditions also significantly differed across all three walking habit groups (*p* = 0.04), and the proportion of health-conscious walkers was more substantial among the subgroups who walked longer. Regarding mental health conditions, the distribution of the subpopulation who suffered from depressive symptoms (CES-D scores ≧ 16) and PTSR (IES-R scores ≧ 25) significantly differed among the three walking durations (*p* = 0.007 and *p* = 0.02, respectively), but psychological pain did not (K6 scores ≧ 9). The proportion of people suffering from depressive symptoms and PTSR were more substantial among the subgroups who walked shorter. Distributions of the other characteristics examined in the study were not significantly different among the three groups (Table 1). In the comparisons of mental health indicators among the three walking duration groups using ANCOVA showed that there were significant association seen in K6 and IES-R scores (*p* = 0.02, *p* = 0.02) but not in CES-D scores (Table 2). Regarding K6 and IES-R, scores were affected not only by walking durations but also by age, gender, and alcohol drinking habits.

**Table 1:** Demographics of the participants

		Number of participants in each demographic category	Number (%) of participants walking≧ 60 min/day	Number (%) of participants walking between 30 and 60 min/day	Number (%) of participants walking < 30 min/day	*p* value
Total number		718	232	259	227	
Age	20–29	101	41 (17.7)	29 (11.2)	31 (13.7)	0.03*
	30–39	108	38 (16.4)	42 (16.2)	28 (12.3)	
	40–49	144	50 (21.6)	54 (20.8)	40 (17.6)	
	50–59	155	54 (23.3)	59 (22.8)	42 (18.5)	
	60–69	123	27 (11.6)	47 (18.1)	49 (22.6)	
	70 ≦	87	22 (9.5)	28 (10.9)	37 (16.3)	
Gender	Male	329	128 (55.2)	119 (45.9)	82 (36.1)	< 0.001**
	Female	389	104 (44.8)	140 (54.1)	145 (63.9)	
The number of family members	1	39	9 (3.9)	14 (5.4)	16 (7.0)	0.57
	2	126	33 (14.2)	47 (18.1)	46 (20.3)	
	3	183	61 (26.3)	69 (26.6)	53 (23.3)	
	4	123	48 (20.7)	36 (13.9)	39 (17.2)	
	5	91	33 (14.2)	31 (12.0)	27 (11.9)	
	6	93	28 (12.1)	36 (13.9)	29 (12.8)	
	7	37	10 (4.3)	17 (6.6)	10 (4.4)	
	8	10	4 (1.7)	4 (1.5)	2 (0.9)	
	9	5	3 (1.3)	2 (0.8)	0 (0.0)	
Residential condition	Living in the same place as before the GEJE	169	62 (26.7)	56 (21.6)	51 (22.5)	0.51
	Living in a different place not due to the disaster	21	6 (2.6)	7 (2.7)	8 (3.5)	
	Post-disaster publicly funded rental accommodation	5	1 (0.4)	3 (1.2)	1 (0.4)	
	Houses of relatives or friends	30	7 (3.0)	14 (5.4)	9 (4.0)	
	Prefabricated post-disaster public housing	74	24 (10.3)	24 (9.3)	26 (11.5)	
	Newly built houses in the relocated high-land area	171	62 (26.7)	61 (23.6)	48 (21.1)	
	Newly built houses outside of the relocated high-land area	175	42 (18.1)	71 (27.4)	62 (27.3)	
	Others	62	23 (9.9)	20 (7.7)	19 (8.4)	
Perceived economic status	Extremely difficult	42	19 (8.2)	14 (5.4)	9 (4.0)	0.41
	Difficult	130	46 (19.8)	41 (15.8)	43 (18.9)	
	A little difficult	215	65 (28.0)	77 (29.7)	73 (32.2)	
	Fine	328	101 (43.5)	125 (48.3)	102 (44.9)	
Alcohol drinking habits	Drinking	273	98 (42.2)	105 (40.5)	70 (30.8)	0.03*
	Non-drinking	397	120 (51.7)	136 (52.5)	141 (62.1)	
Smoking habits	Smoking	138	50 (21.6)	46 (17.8)	42 (18.5)	0.52
	Non-smoking	535	167 (72.0)	197 (76.1)	171 (75.3)	
Walk with health consciousness	Yes	366	113 (48.7)	148 (57.1)	105 (46.3)	0.04*
	No	352	119 (51.3)	111 (42.9)	122 (53.7)	
CES-D	< 16	532	180 (77.6)	201 (77.6)	151 (66.5)	0.007*
	≧ 16	186	52 (22.4)	58 (22.4)	76 (33.5)	
K6	< 9	630	206 (88.8)	233 (90.0)	191 (84.1)	0.13
	≧ 9	88	26 (11.2)	26 (10.0)	36 (15.9)	
IES-R	< 25	576	191 (82.3)	217 (83.8)	168 (74.0)	0.02*
	≧ 25	142	41 (17.7)	42 (16.2)	59 (26.0)	

Distribution of the participants regarding age groups (20–29, 30–39, 40–49, 50–59, 60–69, 70 ≦), gender, number of family members, residential status at the time of the survey [living in the same place as before the Great East Japan Earthquake (GEJE), living in a different place with reasons other than the consequence of the GEJE, post-disaster publicly funded rental accommodation, houses of relatives or friends, prefabricated post-disaster public housing, newly built houses in the relocated high-land area, newly built houses outside of the relocated high-land area, others], economic states (extremely difficult, difficult, a little difficult, fine), alcohol consumption (drinking, non-drinking), smoking status (smoking, non-smoking), walking with health consciousness (yes, no), the Center for Epidemiologic Studies Depression Scale (CES-D; non-depressive: < 16, depressive: ≧ 16), the Kessler Psychological Distress Scale (K6; non-distressed: < 9, distressed: ≧ 9), and the Impact of Event Scale-Revised (IES-R; non-PTSR < 25, PTSR ≧ 25). The distributions of the above parameters were shown for each walking durations subgroup (≧ 60 min/day, between 30 and 60 min/day, < 30 min/day). Differences in the distributions of each parameter among the three walking duration subgroups were evaluated using the Chi-square test

Significance levels: *p < 0.05, **p < 0.01

**Table 2:** The results of ANCOVA to assess the influence of walking durations and covariate factors on the mental health indicators among total participants

Factor	Sum Sq	Df	*F* value	Pr (> *F*)
CES-D				
Age	1161	5	3.41	0.005**
Gender	14	1	0.21	0.65
Alcohol drinking habits	20	1	0.30	0.58
Walking durations	308	2	2.26	0.10
(interaction) Alcohol drinking habits:gender	334	1	4.90	0.03*
K6				
Age	172	5	2.01	0.08
Gender	0.8	1	0.05	0.83
Alcohol drinking habits	11	1	0.66	0.42
Walking durations	133	2	3.89	0.02*
(interaction) Age:gender:walking durations	410	10	2.39	0.009**
IES-R				
Age	3464	5	3.77	0.002**
Gender	44	1	0.24	0.63
Alcohol drinking habits	109	1	0.59	0.44
Walking durations	1497	2	4.07	0.02*
(interaction) Age:gender	2383	5	2.59	0.02*
Alcohol drinking habits:walking durations	1165	2	3.17	0.04*

The table shows the results of the analyses of covariance (ANCOVA) to assess the influence of walking durations, as well as age, gender, and alcohol drinking habits as covariate factors, on the mental health indicators: the Center for Epidemiologic Studies Depression Scale (CES-D), the Kessler Psychological Distress Scale (K6), and the Impact of Event Scale-Revised (IES-R) among total participants

*Sum Sq* sum of squares, *Df* degrees of freedom, *Pr* probability

Significance levels: *p < 0.05, **p < 0.01

### Health-Conscious Walkers

Table 3 illustrates the distributions of characteristics of the three walking duration subgroups among the health-conscious walkers (*N* = 366). The distributions in proportions of each gender and depressed subgroup significantly differed among the three groups (*p* = 0.01 and *p* = 0.007, respectively), and the proportion of females and depressed people were more substantial in the subgroups who walked shorter. The distributions of the other characteristics were not significantly different among the three-walking duration groups. In the comparisons of mental health indicators among the three walking duration groups, ANCOVA showed that there were significant differences in CES-D and K6 scores (*p* = 0.01 and *p* = 0.04) but not in IES-R scores (Table 4). In addition, as subtest, *p* values based on the logistic regression model using CES-D and K6 scores as objective variable and walking durations, age, gender, and alcohol drinking habits as an independent variable were analyzed by Bonferroni multiple comparison corrections. CES-D score was significantly different between long and short walkers (*p* = 0.004). K6 score was not significantly different among the three walking duration groups. Also, there were no significant differences in total IES-R scores and subdomain scores of IES-R among the three walking duration groups (Table 5).

**Table 3:** Demographics of health-conscious walkers

		Number of participants in each demographic category	Number (%) of participants walking≧ 60 min/day	Number (%) of participants walking between 30 and 60 min/day	Number (%) of participants walking < 30 min/day	*p* value
Total Number		366	113	148	105	
Age	20–29	31	12 (10.6)	10 (6.8)	9 (8.6)	0.25
	30–39	43	16 (14.2)	19 (12.8)	8 (7.6)	
	40–49	60	22 (19.5)	21 (14.2)	17 (16.2)	
	50–59	92	32 (28.3)	37 (25.0)	23 (21.9)	
	60–69	84	15 (13.3)	39 (26.4)	30 (28.6)	
	70 ≦	56	16 (14.2)	22 (14.9)	18 (17.1)	
Gender	Male	165	59 (52.2)	71 (48.0)	35 (33.3)	0.01*
	Female	201	54 (47.8)	77 (52.0)	70 (66.7)	
The number of family members	1	21	5 (4.4)	8 (5.4)	8 (7.6)	0.59
	2	75	18 (15.9)	33 (22.3)	24 (22.9)	
	3	87	29 (25.7)	38 (25.7)	20 (19.0)	
	4	56	17 (15.0)	22 (14.9)	17 (16.2)	
	5	52	22 (19.5)	15 (10.1)	15 (14.3)	
	6	47	13 (11.5)	19 (12.8)	15 (14.3)	
	7	16	5 (4.4)	7 (4.7)	4 (3.8)	
	8	7	1 (0.9)	4 (2.7)	2 (1.9)	
	9	2	2 (1.8)	0 (0.0)	0 (0.0)	
Residential condition	Living in the same place as before the GEJE	89	33 (29.2)	35 (23.6)	21 (20.0)	0.27
	Living in a different place not due to the disaster	7	3 (2.7)	0 (0.0)	4 (3.8)	
	Post-disaster publicly funded rental accommodation	2	0 (0.0)	2 (1.4)	0 (0.0)	
	Houses of relatives or friends	14	3 (2.7)	7 (4.7)	4 (3.8)	
	Prefabricated post-disaster public housing	35	7 (6.2)	14 (9.5)	14 (13.3)	
	Newly built houses in the relocated high-land area	91	32 (28.3)	34 (23.0)	25 (23.8)	
	Newly built houses outside of the relocated high-land area	95	24 (21.2)	44 (29.7)	27 (25.7)	
	Others	29	9 (8.0)	10 (6.8)	10 (9.5)	
Perceived economic status	Extremely difficult	20	7 (6.2)	7 (4.7)	6 (5.7)	0.98
	Difficult	59	19 (16.8)	23 (15.5)	17 (16.2)	
	A little difficult	106	29 (25.7)	45 (30.4)	32 (30.5)	
	Fine	179	58 (51.3)	71 (48.0)	50 (47.6)	
Alcohol drinking habits	Drinking	140	45 (39.8)	61 (41.2)	34 (32.4)	0.45
	Non-drinking	190	58 (51.3)	74 (50.0)	58 (55.2)	
Smoking habits	Smoking	55	18 (15.9)	21 (14.2)	16 (15.2)	0.93
	Non-smoking	290	89 (78.8)	118 (79.7)	83 (79.0)	
CES-D	< 16	279	96 (85.0)	113 (76.4)	70 (66.7)	0.007**
	≧ 16	87	17 (15.0)	35 (23.6)	35 (33.3)	
K6	< 9	328	106 (93.8)	133 (89.9)	89 (84.8)	0.09
	≧ 9	38	7 (6.2)	15 (10.1)	16 (15.2)	
IES-R	< 25	294	96 (85.0)	120 (81.1)	78 (74.3)	0.14
	≧ 25	72	17 (15.0)	28 (18.9)	27 (25.7)	

Distribution of walker with health-conscious group regarding age groups (20–29, 30–39, 40–49, 50–59, 60–69, ≦ 70), gender, the number of family members, residential status at the time of the survey [living in the same place as before the Great East Japan Earthquake (GEJE), living in a different place with reasons other than the consequence of the GEJE, post-disaster publicly funded rental accommodation, houses of relatives or friends, prefabricated post-disaster public housing, newly built houses in the relocated high-land area, newly built houses outside of the relocated high-land area, others], economic states (extremely difficult, difficult, a little difficult, fine), alcohol consumption (drinking, non-drinking), smoking status (smoking, non-smoking), the Center for Epidemiologic Studies Depression Scale (CES-D; non-depressive: < 16, depressive: ≧ 16), the Kessler Psychological Distress Scale (K6; non-distressed: < 9, distressed: ≧ 9), and the Impact of Event Scale-Revised (IES-R; non-PTSR < 25, PTSR ≧ 25). The distributions of the above parameters were shown for each walking durations subgroup (≧ 60 min/day, between 30 and 60 min/day, < 30 min/day). Differences in the distributions of each parameter among the three walking duration subgroups were evaluated using the Chi-square test

Significance levels: *p < 0.05, **p < 0.01

**Table 4:** The results of ANCOVA to assess the influence of walking durations and covariate factors on the mental health indicators among health-conscious walkers

	Sum Sq	Df	*F* value	Pr (> *F*)
CES-D				
Age	534	5	1.74	0.1
Gender	7	1	0.12	0.73
Alcohol drinking habits	19	1	0.30	0.58
Walking durations	553	2	4.49	0.01*
K6				
Age	111	5	1.54	0.18
Gender	5	1	0.32	0.57
Alcohol drinking habits	4	1	0.26	0.61
Walking durations	90	2	3.14	0.04*
(interaction) Age:alcohol drinking habits	172	5	2.39	0.04*
IES-R				
Age	1943	5	2.48	0.03*
Gender	5	1	0.03	0.86
Alcohol drinking habits	361	1	2.31	0.13
Walking durations	614	2	1.96	0.14
(interaction) Age:walking durations	3090	10	1.98	0.04*

The table shows the results of the analyses of covariance (ANCOVA) to assess the influence of walking durations, as well as age, gender, and alcohol drinking habits as covariate factors, on the mental health indicators: the Center for Epidemiologic Studies Depression Scale (CES-D), the Kessler Psychological Distress Scale (K6), and the Impact of Event Scale-Revised (IES-R) among health-conscious walkers

*Sum Sq* sum of squares, *Df* degrees of freedom, *Pr* probability

Significance levels: *p < 0.05, **p < 0.01

**Table 5:** The results of ANCOVA to assess the influence of walking durations and covariate factors on the IES-R subdomain indicators among health-conscious walkers

Factor	Sum Sq	Df	*F* value	Pr (> F)
Re-experiencing				
Age	338	5	2.86	0.02*
Gender	1.3	1	0.05	0.82
Alcohol drinking habits	54	1	2.30	0.13
Walking durations	91	2	1.91	0.15
(interaction) Age:walking durations	528	5	2.23	0.02*
Avoidance				
Age	386	5	3.00	0.01*
Gender	10	1	0.37	0.54
Alcohol drinking habits	46	1	1.80	0.18
Walking durations	83	2	1.60	0.2
Hyperarousal				
Age	75	5	1.14	0.34
Gender	4	1	0.29	0.59
Alcohol drinking habits	23	1	1.76	0.19
Walking durations	40	2	1.53	0.22
(interaction) Age:walking durations	250	10	1.90	0.04*

The table shows the results of the analyses of covariance (ANCOVA) to assess the influence of walking durations, as well as age, gender, and alcohol drinking habits as covariate factors, on the Impact of Event Scale-Revised (IES-R) subdomain indicators: re-experiencing, avoidance, and hyperarousal among health-conscious walkers

*Sum Sq* sum of squares, *Df* degrees of freedom, *Pr* probability

Significance levels: *p < 0.05, **p < 0.01

### Non-Health-Conscious Walkers

Table 6 illustrates the distributions of characteristics of the three walking duration subgroups among the non-health-conscious walkers (*N* = 352). The distributions in proportions of subgroups regarding age, gender, and alcohol drinking habits significantly differed among the three walking duration groups (*p* = 0.03, *p* = 0.007, and *p* = 0.04, respectively), and the proportion of elderlies, female, and alcohol drinkers were more substantial among the subgroup with shorter walking durations. In the comparisons of mental health indicators among the three walking duration groups using ANCOVA showed that there were no significant differences in CES-D, K6, and IES-R scores (Table 7). In comparisons of IES-R subdomain scores, there were significant differences in avoidance symptoms (*p* = 0.01), but not in re-experiencing and hyperarousal symptoms, among the three walking duration groups of non-health-conscious walkers (Table 8). In addition, the logistic regression analysis was done using avoidance symptoms as objective variable and walking durations, age, gender, and alcohol drinking habits as an independent variable followed by the Bonferroni multiple comparison correction. Avoidance symptoms were significantly different between moderate and short walkers (*p* = 0.002).

**Table 6:** Demographics of non-health-conscious walkers

		Number of participants in each demographic category	Number (%) of participants walking≧ 60 min/day	Number (%) of participants walking between 30 and 60 min/day	Number (%) of participants walking < 30 min/day	*p* value
Total number		352	119	111	122	
Age	20–29	70	29 (24.4)	19 (17.1)	22 (18.0)	0.03*
	30–39	65	22 (18.5)	23 (20.7)	20 (16.4)	
	40–49	84	28 (23.5)	33 (29.7)	23 (18.9)	
	50–59	63	22 (18.5)	22 (19.8)	19 (15.6)	
	60–69	39	12 (10.1)	8 (7.2)	19 (15.6)	
	70 ≦	31	6 (5.0)	6 (5.4)	19 (15.6)	
Gender	Male	164	69 (58.0)	48 (43.2)	47 (38.5)	0.007**
	Female	188	50 (42.0)	63 (56.8)	75 (61.5)	
The number of family members	1	18	4 (3.4)	6 (5.4)	8 (6.6)	0.17
	2	51	15 (12.6)	14 (12.6)	22 (18.0)	
	3	96	32 (26.9)	31 (27.9)	33 (27.0)	
	4	67	31 (26.1)	14 (12.6)	22 (18.0)	
	5	39	11 (9.2)	16 (14.4)	12 (9.8)	
	6	46	15 (12.6)	17 (15.3)	14 (11.5)	
	7	21	5 (4.2)	10 (9.0)	6 (4.9)	
	8	3	3 (2.5)	0 (0.0)	0 (0.0)	
	9	3	1 (0.8)	2 (1.8)	0 (0.0)	
Residential condition	Living in the same place as before the GEJE	80	29 (24.4)	21 (18.9)	30 (24.6)	0.47
	Living in a different place not due to the disaster	14	3 (2.5)	7 (6.3)	4 (3.3)	
	Post-disaster publicly funded rental accommodation	3	1 (0.8)	1 (0.9)	1 (0.8)	
	Houses of relatives or friends	16	4 (3.4)	7 (6.3)	5 (4.1)	
	Prefabricated post-disaster public housing	39	17 (14.3)	10 (9.0)	12 (9.8)	
	Newly built houses in the relocated high-land area	80	30 (25.2)	27 (24.3)	23 (18.9)	
	Newly built houses outside of the relocated high-land area	80	18 (15.1)	27 (24.3)	35 (28.7)	
	Others	33	14 (11.8)	10 (9.0)	9 (7.4)	
Perceived economic status	Extremely difficult	22	12 (10.1)	7 (6.3)	3 (2.5)	0.15
	Difficult	71	27 (22.7)	18 (16.2)	26 (21.3)	
	A little difficult	109	36 (30.3)	32 (28.8)	41 (33.6)	
	Fine	149	43 (36.1)	54 (48.6)	52 (42.6)	
Alcohol drinking habits	Drinking	133	53 (44.5)	44 (39.6)	36 (29.5)	0.04*
	Non-drinking	207	62 (52.1)	62 (55.9)	83 (68.0)	
Smoking habits	Smoking	83	32 (26.9)	25 (22.5)	26 (21.3)	0.52
	Non-smoking	245	78 (65.5)	79 (71.2)	88 (72.1)	
CES-D	< 16	253	84 (70.6)	88 (79.3)	81 (66.4)	0.09
	≧ 16	99	35 (29.4)	23 (20.7)	41 (33.6)	
K6	< 9	302	100 (84.0)	100 (90.1)	102 (83.6)	0.29
	≧ 9	50	19 (16.0)	11 (9.9)	20 (16.4)	
IES-R	< 25	282	95 (79.8)	97 (87.4)	90 (73.8)	0.03*
	≧ 25	70	24 (20.2)	14 (12.6)	32 (26.2)	

Distribution of walker without health-consciousness group regarding age groups (20–29, 30–39, 40–49, 50–59, 60–69, ≧ 70), gender, number of family members, residential status at the time of the survey [living in the same place as before the Great East Japan Earthquake (GEJE), living in a different place with reasons other than the consequence of the GEJE, post-disaster publicly funded rental accommodation, houses of relatives or friends, prefabricated post-disaster public housing, newly built houses in the relocated high-land area, newly built houses outside of the relocated high-land area, others], economic states (extremely difficult, difficult, a little difficult, fine), alcohol consumption (drinking, non-drinking), smoking status (smoking, non-smoking), the Center for Epidemiologic Studies Depression Scale (CES-D; non-depressive: < 16, depressive: ≧ 16), the Kessler Psychological Distress Scale (K6; non-distressed: < 9, distressed: ≧ 9), and the Impact of Event Scale-Revised (IES-R; non-PTSR < 25, PTSR ≧ 25). The distributions of the above parameters were shown for each walking durations subgroup (≧ 60 min/day, between 30 and 60 min/day, < 30 min/day). Differences in the distributions of each parameter among the three walking duration subgroups were evaluated using the Chi-square test

Significant levels: *p < 0.05, **p < 0.01

**Table 7:** The results of ANCOVA to assess the influence of walking durations and covariate factors on the mental health indicators among non-health-conscious walkers

Factor	Sum Sq	Df	*F* value	Pr (> *F*)
CES-D				
Age	705	5	2.04	0.07
Gender	132	1	1.91	0.17
Alcohol drinking habits	75	1	1.093	0.3
Walking durations	337	2	2.44	0.09
(interaction) Age:gender	950	5	2.75	0.02*
Alcohol drinking habits:gender	398	1	5.77	0.02*
Age:alcohol drinking habits:gender	1112	5	3.22	0.008**
Age:gender:walking durations	2112	9	3.40	< 0.001***
K6				
Age	78	5	0.82	0.53
Gender	13	1	0.70	0.4
Alcohol drinking habits	6	1	0.30	0.59
Walking durations	75	2	1.99	0.14
(interaction) Age:gender:walking durations	662	9	3.91	< 0.001***
IES-R				
Age	1304	5	1.32	0.26
Gender	395	1	2.00	0.16
Alcohol drinking habits	781	1	3.95	0.047*
Walking durations	1170	2	2.96	0.054
(interaction) Age:gender	4534	5	4.59	< 0.001***

The table shows the results of the analyses of covariance (ANCOVA) to assess the influence of walking durations, as well as age, gender, and alcohol drinking habits as covariate factors, on the mental health indicators: the Center for Epidemiologic Studies Depression Scale (CES-D), the Kessler Psychological Distress Scale (K6), and the Impact of Event Scale-Revised (IES-R) among non-health-conscious walkers

*Sum Sq* sum of squares, *Df* degrees of freedom, *Pr* probability

Significance levels: *p < 0.05, **p < 0.01, ***p < 0.001

**Table 8:** The results of ANCOVA to assess the influence of walking durations and covariate factors on the IES-R subdomain indicators among non-health-conscious walkers

Factor	Sum Sq	Df	*F* value	Pr (> *F*)
Re-experiencing				
Age	224	5	1.63	0.15
Gender	66	1	2.39	0.12
Alcohol drinking habits	107	1	3.88	0.049*
Walking durations	119	2	2.16	0.12
(interaction) Age:gender	614	5	4.46	< 0.001***
Avoidance				
Age	264	5	1.80	0.11
Gender	45	1	1.54	0.22
Alcohol drinking habits	137	1	4.65	0.03*
Walking durations	251	2	4.27	0.01*
(interaction) Age:gender	463	5	3.15	0.009**
Hyperarousal				
Age	48	5	0.53	0.75
Gender	26	1	1.41	0.24
Alcohol drinking habits	35	1	1.94	0.17
Walking durations	77	2	2.14	0.12
(interaction) Age:gender	474	5	5.24	< 0.001***
Age:gender:walking durations	357	9	2.19	0.02*

The table shows the results of the analyses of covariance (ANCOVA) to assess the influence of walking durations, as well as age, gender, and alcohol drinking habits as covariate factors, on the Impact of Event Scale-Revised (IES-R) subdomain indicators: re-experiencing, avoidance, and hyperarousal among non-health-conscious walkers

*Sum Sq* sum of squares, *Df* degrees of freedom, *Pr* probability

Significance levels: *p < 0.05, **p < 0.01, ***p < 0.001

The levels of emotional pain when seeing signs relevant to a disaster or landscape of the sea in the living environment after the disaster were also compared using ANCOVA. There was no significant difference in the level of emotional pain among the three walking duration groups (Table 9).

**Table 9:** The results of ANCOVA to assess the influence of walking durations and covariate factors on the indicator of emotional pain among total participants

Factor	Sum Sq	Df	*F* value	Pr (> *F*)
Emotional pain				
Age	15.97	5	3.49	0.004**
Gender	0.49	1	0.54	0.46
Alcohol drinking habits	0.48	1	0.53	0.47
Walking durations	1.65	2	0.90	0.41

The table shows the results of the analyses of covariance (ANCOVA) to assess the influence of walking durations, as well as age, gender, and alcohol drinking habits as covariate factors, on the indicator of feeling emotional pain by seeing signs related to a disaster or the landscape of the sea (emotional pain) among total participants

*Sum Sq* sum of squares, *Df* degrees of freedom, *Pr* probability

Significance level: **p < 0.01

## Discussion

The current study indicated a significant negative association between walking durations and depressive symptoms in post-disaster settings, which endorsed the positive effect of walking on mental health conditions seen in previous studies conducted in ordinary settings [16–22]. Multi-level meta-analyses have shown that walking, as well as other physical activities, is positively associated with mental health [16], and several interventions that encourage moderate levels of walking or increased physical activities have been shown to reduce depressive symptoms [17–19]. A national study of young adults revealed that taking more than 7500 steps per day was associated with fewer depressive symptoms [20].

However, these prior studies have rarely differentiated between subjects who walked for health-conscious reasons versus those who walked for other purposes. This question is crucial to explore as the intention behind walking may impact the overall association between walking and improvement of mental health conditions. The current study provided evidence to suggest that only when people walk with a health-conscious mindset can walking have positively affected to improving their depressive symptoms. There was a similar study based on adults aged 60–64 years old showing that leisure-time physical activities and walking for pleasure were positively correlated with good mental health conditions, whereas total levels of free-living physical activities were not [27]. The previous study differs from the current investigation in several crucial regards. Whereas the previous study investigates merely the elderly population, subjects of the current study had a more extensive range of ages, and whereas target physical activities of the previous study included a wide range of activities, the current study focused solely on walking. Also, whereas the previous study classified the purpose of activities and walking into either for pleasure or not, the current study classified the purpose of walking into either they walked for maintaining good health condition or not. The two purposes of walking, for pleasure and health, may not be mutually exclusive but rather may overlap in many cases, and walking with either or both of the purposes may be associated with good mental health conditions.

Another interesting study investigated the impact of the placebo effect on one’s perceived impact of physical activity on overall health. More specifically, it illustrated that individuals who were told that their daily work was a good source of exercise (cleaning hotel rooms) perceived themselves to be getting significantly more exercise than before they were informed of this exercise benefit. As a result, compared with the control group, the informed group later showed a decrease in weight, blood pressure, body fat, waist-to-hip ratio, and body mass index [38]. This result suggests that placebo effects can significantly influence physical health outcomes. However, it is also possible that participants with these mindsets have changed the quality and quantity of their engaged physical activity by altering the exercise frequency, pace (intensity, time), and manner of their physical movements (type) [25].

Another study examined the physical and psychological consequences of undergraduate students who were placed in three different exercise groups: no aerobic exercise, aerobic exercise only, or aerobic exercise with deliberate manipulation of their expectations (i.e., told of benefits) [39]. Ultimately, they found that individuals placed in both the aerobic exercise-only group and the aerobic exercise with deliberate manipulation of expectations group showed significant improvements in cardiovascular fitness. Additionally, the aerobic exercise with the manipulation of expectations group was found to have significant improvements in well-being and self-esteem. While it was not certain that the belief in the positive effects of exercise positively affected physical health outcomes, the study suggested that the belief in the positive effects of exercise may improve mental health outcomes along with the physical health outcomes.

Thus, our results suggest that altering one’s mindset to believe in the positive health outcomes of exercise, similar to the placebo effect seen in past research, may be associated with improved mental health. However, caution is needed because our study is a cross-sectional study. Instead of manipulating the motivation or mindsets of our participants, we have only examined the associations between the intention behind walking and their mental health and well-being. In previous studies, motivation and optimism were negatively associated with mental health conditions in general [28–30] and PTSR in communities affected by a disaster [40–42]. Our data might reflect less motivation and optimism in maintaining good health, probably due to poor mental health conditions.

Previous studies have reported that increased physical activity can lead to reduced PTSD symptoms [43, 44], but our study provides initial data to suggest a relationship between PTSR symptoms and health consciousness, specifically during walking behaviors. When walking was done with the intention of maintaining good health, longer walking durations were not associated with PTSR, unlike its association with depressive symptoms. These results suggest that walking habits may affect the symptoms of depression and PTSR in different ways.

On the other hand, our study suggests that people who walk less than 30 min/day among non-health-conscious walkers might have been somehow influenced by avoidance symptoms. Participants with avoidance symptoms of PTSR may deliberately avoid going out to walk, ultimately worsening their general health conditions, including depressive symptoms.

Considering that there are many signs relevant to a disaster in the living environment of these participants, for example, signs of the inundated area of the GEJE or prior evacuation points, the hypothesis that emotional pain from seeing reminders of post-disaster environments is associated with less walking duration was tested. However, this hypothesis was ultimately not supported by the current study. It may be necessary to intervene with people affected by disasters to increase health-conscious physical activities; however, the effective solution may not be to eliminate environmental triggers.

There were several limitations to this study. First, this study was a cross-sectional study based on the questionnaire survey conducted 7 years after the GEJE; therefore, a causal relationship between walking habit and mental health condition was not determined. Second, more detailed assessments of the manner of walking, such as intensity and posture of walking, were not conducted, and information regarding exercises other than walking was not taken into consideration. The reproducibility of the findings, along with the above issues, needs to be examined further in future studies with independent populations.

## Conclusion

The current study provides initial evidence to support a negative association between walking durations and depressive state, but not PTSR, only among individuals who walked with a health-conscious mindset. Walking durations were negatively associated with avoidance symptoms among non-health-conscious walkers in the community affected by the GEJE, indicating that the disaster may have had a long-lasting impact on walking habits. Emphasizing the health benefits of walking for individuals recovering from a disaster may be beneficial for improving the mental health conditions of affected individuals.

## Data Availability

Detailed data are available for access upon request. In those cases, some of the information will be excluded from the raw data to protect personal information.

## Abbreviations

GEJE: The Great East Japan Earthquake
PTSR: Posttraumatic stress reaction
K6: The Kessler Psychological Distress Scale K-6 items
CES-D: The Center for Epidemiologic Studies Depression Scale
IES-R: The Impact of Event Scale-Revised
